# On subcellular distribution of the zinc finger 469 protein (ZNF469) and observed discrepancy in the localization of endogenous and overexpressed ZNF469

**DOI:** 10.1002/2211-5463.70034

**Published:** 2025-03-29

**Authors:** Anne Elisabeth Christensen Mellgren, Ileana Cristea, Thomas Stevenson, Endy Spriet, Per Morten Knappskog, Stig Ove Bøe, Harald Kranz, Sushma N. Grellscheid, Eyvind Rødahl

**Affiliations:** ^1^ Department of Clinical Medicine University of Bergen Norway; ^2^ Department of Ophthalmology Haukeland University Hospital Norway; ^3^ Computational Biology Unit and Department of Biomedicine University of Bergen Norway; ^4^ Molecular Imaging Center, Department of Biomedicine University of Bergen Norway; ^5^ Department of Medical Genetics Haukeland University Hospital Bergen Norway; ^6^ Department of Clinical Science University of Bergen Norway; ^7^ Department of Microbiology Oslo University Hospital Norway; ^8^ Gen‐H Genetic Engineering Heidelberg GmbH Heidelberg Germany

**Keywords:** biomolecular condensates, intrinsically disordered regions, midbody, nuclear speckles, proteasomes, stress granules

## Abstract

The zinc finger 469 gene (*ZNF469*) is a single‐exon gene predicted to encode a protein of 3953 amino acids. Despite pathogenic *ZNF469* variants being associated with Brittle Cornea Syndrome (BCS), relatively little is known about ZNF469 beyond its participation in regulating the expression of genes encoding extracellular matrix proteins. In this study, we examined the expression and intracellular localization of ZNF469 in different cell lines. The level of ZNF469 mRNA varied from low levels in HEK293 cells to high levels in HeLa cells and primary fibroblasts. Antibodies against ZNF469 reacted among others with a protein of approximately 400 kDa in immunoblot analysis, which was mainly present in the insoluble fraction of the cytoplasm. Immunofluorescence analysis of interphase cells showed small cytoplasmic puncta and weak nuclear staining. In dividing HeLa cells, the antibodies recognized foci that also stained for proteasomes. In transfected cells, ZNF469 was observed mainly in foci resembling nuclear speckles in interphase and at the midbody during mitosis. The nuclear foci showed overlapping staining with proteasomes. In live cell imaging, liquid‐like properties of the nuclear foci were recorded as they changed shape and position and occasionally fused with each other. During stress granule formation, cytoplasmic foci showed overlapping staining with G3BP1. Finally, *in silico* analysis revealed large intrinsically disordered regions with multiple low complexity domains in ZNF469. Our data indicate that ZNF469 forms aggregates possibly as biomolecular condensates when overexpressed. However, care must be taken when analyzing the intracellular distribution of ZNF469 due to the discrepancy in the localization of endogenous ZNF469 and overexpressed ZNF469 in transfected cells.

## Graphical abstract

ZNF469 regulates the expression of genes encoding extracellular matrix proteins. Endogenous ZNF469 is predominantly cytoplasmic, while in transfected cells, it forms aggregates reminiscent of biomolecular condensates, located mainly in the nucleus. These condensates exhibit overlapping staining with proteasomes and are also associated with the mitotic spindle, suggesting ZNF469 might be involved in a complex interplay of cellular processes.

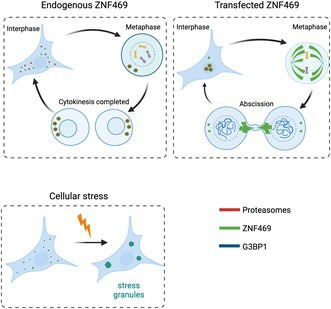

**Abbreviations**
AcGFP: *Aequorea coerulescens* green fluorescent protein 1
ATCC: American Type Culture Collection
ATPIF1: ATP synthase inhibitory factor subunit 1
BAC: bacterial artificial chromosome
BCS: brittle cornea syndrome
CAMP: chromosome alignment maintaining phosphoprotein
D2P2: database of disordered protein prediction
DAPI: 4′,6‐diamidino‐2‐phenylindole
DMEM: Dulbecco's modified Eagle's medium
ECM: extracellular matrix
ER: endoplasmic reticulum
G3BP1: Ras‐GTPase‐activating protein SH3‐domain‐binding protein (G3BP stress granule assembly factor 1)
GAPDH: glyceraldehyde‐3‐phosphate dehydrogenase
GM130: golgi matrix protein 130
HEK: human embryonic kidney
HeLa: Henrietta Lacks
HP1α: heterochromatin protein 1 alpha
HPRT1: hypoxanthine phosphoribosyltransferase 1
HRP: horseradish peroxidase
LAMP1: lysosomal‐associated membrane protein 1
MobiDB: A Database of Protein Disorder and Mobility Annotations
MoRFs: molecular recognition features
NES: nuclear export signal
NLS: nuclear localization signal
PCNA: proliferating cell nuclear antigen
PML: promyelocytic leukemia
PRDM5: PR/SET‐domain 5
RIDAO: rapid intrinsic disorder analysis online
RPE‐1: hTERT‐immortalized Retinal Pigment Epithelial cells
SMART: A Simple Modular Architecture Research Tool
SPC25: signal peptidase complex accessory subunit 25
SUMO1: small ubiquitin‐related modifier 1
TOMM20: translocase of outer mitochondrial membrane 20
TRF2: telomeric repeat‐binding factor 2
U2OS: human osteosarcoma cell line
ZNF: zinc finger

The zinc finger 469 gene (*ZNF469*) (https://www.ncbi.nlm.nih.gov/gene/84627) is a single‐exon gene [1] located on chromosome 16q24.2. The full‐length mRNA is predicted to encode a large protein of 3953 amino acids with eight potential Cys2His2 (C2H2) zinc finger domains located mainly in the C‐terminal part of the protein (Fig. S1).

Pathogenic variants in *ZNF469* are associated with Brittle Cornea Syndrome (BCS1; OMIM 229200), a rare, autosomal recessive connective tissue disorder. A similar phenotype is seen in individuals with pathogenic variants in the PR/SET‐Domain 5 (*PRDM5*) gene (BCS2; OMIM 614170), [2, 3, 4] (summary of reported *ZNF469* variants in [5]). Among the typical manifestations are kyphoscoliosis, hearing impairment, joint hypermobility, skin hyperelasticity, decreased bone mineral density, and dental abnormalities. The ocular features include extreme thinning of the cornea, sometimes leading to spontaneous rupture, accompanied by malformations like keratoconus, keratoglobus, and blue sclera.

Genome wide association studies have revealed that common variants near *ZNF469* contribute to variation in central corneal thickness [6, 7]. Conflicting evidence has been reported for the role of ZNF469 in the development of non‐syndromic keratoconus [8, 9, 10].

Studies of patient fibroblasts and fibroblasts from mice and zebrafish lacking ZNF469 have demonstrated a change in mRNA levels of extracellular matrix (ECM) genes, suggesting a role for ZNF469 in transcriptional regulation of ECM genes [1, 4, 5, 11]. In their zebrafish model, Bao *et al*. also observed increased expression of genes encoding proteasomal proteins [11]. A role for ZNF469 in regulating the expression of profibrotic genes was recently reported in hepatic stellate cells [12].

ZNF469 mRNA is present in most human tissues including the cornea and sclera, [2] with the highest levels detected in the ovary and brain (https://www.proteinatlas.org/search/ZNF469). Considerable variation in the level of ZNF469 mRNA in different cell lines has been observed with relatively high levels in HeLa cells and fibroblasts and low/undetectable levels in HEK293 cells. Intracellularly, the ZNF469 protein has been assigned to the nucleus but has also been found at cell junctions (https://www.proteinatlas.org/search/ZNF469). In transient transfection experiments, ZNF469 has been detected in the nucleus [12].

The aim of the present study was to examine the expression and intracellular localization of ZNF469 in greater detail using anti‐ZNF469 antibodies and plasmids expressing ZNF469 with and without a GFP tag.

## Materials and methods

### *In silico* analysis

Functional domains were identified using the InterPro (https://www.ebi.ac.uk/interpro/) [13] and SMART (http://smart.embl.de) database [14]. Intrinsically disordered regions were mapped using MobiDB (https://mobidb.org) [15], Database of Disordered Protein Prediction (D^2^P^2^) (http://d2p2.pro/) [16] and Rapid Intrinsic Disorder Analysis Online (RIDAO) (https://ridao.app) [17]. Classical nuclear localization signals were identified using cNLS Mapper (https://nls‐mapper.iab.keio.ac.jp/) [18]. A search for nuclear export signals was performed using LocNES (http://prodata.swmed.edu/LocNES/LocNES.php) [19].

### Cells and tissues

Human embryonic kidney (HEK)293 cells (# CRL‐1573™; ATCC, Manassas, VA, USA), HeLa cells (#CCl‐2™; ATCC), SV40 immortalized human fibroblasts GM847 (#GM00847; Coriell Institute for Medical Research, Camden, NJ, USA), hTERT‐immortalized retinal pigment epithelial cells (RPE‐1) (#CRL‐4000; ATCC), wild‐type U2OS and ΔΔG3BP1/2 knock‐out U2OS cells stably transduced with mCherry‐G3BP1 (kindly provided by N. Kedersha, Brigham and Women's Hospital, Harvard Medical School, Boston, MA, USA) [20, 21] were cultured in Dulbecco's Modified Eagle's Medium (DMEM) containing 4.5 g·L^−1^ glucose (Lonza Bioscience, Basel, Switzerland) supplemented with 10% fetal calf serum, 2 mm L‐Glutamine, 100 U·mL^−1^ penicillin, and 0.1 mg·mL^−1^ streptomycin. GM847 cells [22] are a subclone of LN‐SV cells [23] that exhibit alternative lengthening of telomeres and express elevated levels of Promyelocytic Leukemia (PML) bodies.

Primary human skin fibroblasts were grown from punch biopsies from healthy individuals [24]. Approval for use of human material was obtained from the Regional Committee for Health and Research Ethics of Western Norway (IRB# 00001872) (ref. no. 3.2007.1368). Written informed consent was obtained from the healthy individuals. The study thus adhered to the Tenets of the Declaration of Helsinki.

### Antibodies

The anti‐ZNF469 antibodies used in the present study were affinity purified rabbit antibodies against a peptide corresponding to amino acids 1659–1749 of ZNF469 (#PA5‐67072; ThermoFisher Scientific, Waltham, MA, USA). The supplier has later replaced this antibody with a new antibody directed against the same amino acids (#PA5‐145175).

Mouse monoclonal antibodies against the following proteins were used in double labeling immunofluorescence experiments: sc‐35 (kind gift from Prof. Karl‐Henning Kalland, University of Bergen), PML (#sc‐966; Santa Cruz Biotechnology, Dallas, TX, USA), TRF2 (#05‐521; Merck KGaA, Darmstadt, Germany), SUMO‐1 (#33–2400, ThermoFisher Scientific), U1 snRNP (#SAB4200188, Merck), acetylated tubulin (#T6793, Merck), α‐tubulin (#3873; Cell Signaling Technology, Danvers, MA, USA), β‐tubulin (#T4026; Merck), SPC25 (kindly provided by Stephen High, University of Manchester, UK), GM130 (#610823; BD Biosciences, Franklin Lakes, NJ, USA), TOMM20 (#WH0009804M1, Merck), ATPIF1 (#A‐21355; ThermoFisher Scientific), LAMP1 (#sc‐18 821, Santa Cruz Biotechnology), proteasome 19S S5A/ASF (#ab20239; Abcam, Cambridge, UK), proteasome 20S α1 + 2 + 3 + 5 + 6 + 7 (#ab 22 674; Abcam), and G3BP1 (#sc‐365 338; Santa Cruz Biotechnology). Rabbit polyclonal antibodies against SUMO1 (#4930; Cell Signaling Technology), HP1α (#2616; Cell Signaling Technology), PML (#sc‐5621; Santa Cruz Biotechnology), coilin (#sc‐32 860; Santa Cruz Biotechnology), and PCNA (#RB‐9055‐P; ThermoFisher Scientific) were used in immunofluorescence analysis of transfected cells only. Rabbit antibodies against GAPDH (#G9545; Merck), β‐tubulin (#2146; Cell Signaling Technology), Na^+^/K^+^‐ATPase (#ab76020; Abcam), and HP1α (#2616; Cell Signaling Technology) were used in immunoblots. Secondary antibodies for immunofluorescence analysis were Alexa Fluor 594 conjugated affinity purified (Fab)_2_ fragment goat anti‐mouse (#115‐586‐146) or goat anti‐rabbit IgG (#111‐586‐144), and Alexa Fluor 488 conjugated affinity purified (Fab)_2_ fragment goat anti‐rabbit IgG (#111‐546‐144) (Jackson ImmunoResearch, West Grove, PA, USA). For immunoblots, HRP‐conjugated goat anti‐rabbit IgG antibodies (#7074; Cell Signaling Technology) were used.

### Plasmids

A BAC clone containing *ZNF469* from the Caltech‐D human BAC library was obtained from Invitrogen (#CTD‐2589B6; ThermoFisher Scientific) (the BAC clone is unfortunately not available from this supplier after 1 Oct 2022). Full‐length *ZNF469* was inserted into pAcGFP1‐N1 (Takara Bio, San Jose, CA, USA) and pCMVTNT (Promega, Madison, WI, USA) using homologous recombination by Gene Bridges (Gene Bridges, Heidelberg, Germany). A Kozak sequence was added to the 5′ end of *ZNF469* [25]. In pAcGFP1‐N1, a 10 amino acid (Gly4Ser)2 linker was inserted between *ZNF469* and the 5' end of the *Aequorea coerulescens* green fluorescent protein 1 gene. The plasmids were sequenced using Sanger sequencing. The cloned *ZNF469* represents the complete ZNF469 DNA sequence, allowing for the expression of an mRNA predicted to encode the 3953 amino acids isoform with a predicted molecular weight of 413 kDa. See https://www.ensembl.org/Homo_sapiens/protview?peptide=ENSP00000456500 and https://www.ncbi.nlm.nih.gov/protein/NP_001354553.1.

### Quantitative PCR (TaqMan analysis)

Total RNA was extracted from HEK293, HeLa, RPE‐1, U2OS cells and primary human fibroblasts using the RNeasy Mini Kit (#74104; Qiagen, Germany) according to the manufacturer's instructions. In addition, DNA digestion was performed using DNase I. RNA concentration was assessed using a Nanodrop spectrophotometer. cDNA was synthesized from 1 to 2.5 μg of total RNA using SuperScript™ VILO™ Master Mix (#11755050; ThermoFisher), following the manufacturer's protocol. TaqMan probes for the target gene ZNF469 (reporter dye FAM, #Hs00611441_s1) and the housekeeping gene HPRT1 (reporter dye VIC, #4326321E) were purchased from ThermoFisher. The PCR reaction was performed in a total volume of 10 μL containing 5 μL of TaqMan Universal Master Mix, 250 nm of each TaqMan probe, and 1 μL of cDNA. The cycling conditions included an initial activation at 50 °C for 2 min and denaturation at 95 °C for 10 min, followed by 40 cycles of 95 °C for 15 s and 60 °C for 1 min. Quantitative results were analyzed using the ΔΔCt method, normalizing to the reference gene HPRT1. Data analysis was conducted using the GraphPad Prism software (version 9.5.1). Each assay included no‐template controls. Cells were harvested from three separate dishes. RNA from each dish was subjected to three separate PCR reactions resulting in a total of nine replicates for each cell line.

### Transfection with plasmid DNA

For transient transfection of HEK293, HeLa, GM847, and U2OS cells, the cells were grown on poly‐L‐lysine coated coverslips (0.1 mg·mL^−1^ poly‐L‐lysine (Merck) was used for coating) to approximately 50% confluency. Except for U2OS cells, the cells were transfected with plasmid DNA using FuGENE HD (Promega). To each well in a 12‐well plate, 1 mL of cell culture medium, 2 μg plasmid DNA, and 6 μL FuGENE in 100 μL Opti‐MEM I (ThermoFisher Scientific) were added. U2OS cells were transfected using Lipofectamine 3000 (ThermoFisher Scientific) as recommended by the manufacturer. The coverslips were fixed in 4% paraformaldehyde in 0.1 m phosphate buffer pH 7.5 48 h after the transfection and processed for immunofluorescence analysis using antibodies against marker proteins.

Stably transfected HEK293 cells were generated by incubating the cells in 0.5 mg·mL^−1^ geneticin (G418) (Merck) starting on the second day after transfection. Medium containing G418 was changed every 3–4 days.

### Subcellular fractionation

HEK293 cells were resuspended in cold homogenization buffer (10 mm HEPES buffer pH 7.4, 50 mm sucrose, 1 mm PMSF, 1 μg·mL^−1^ aprotinin, and complete protease inhibitors (Roche Diagnostics)), and homogenized using 8 μm clearance balls (Isobiotec, Heidelberg, Germany) followed by centrifugation as described previously [26]. The suspension was first centrifuged at 2000 ***g***, 4 °C for 10 min. The pellet (nuclear fraction) was resuspended in homogenization buffer. The supernatant (cytoplasmic fraction) was centrifuged at 100 000 ***g***, 4 °C for 60 min. The resulting supernatant represents the soluble cytosol fraction. The pellet (insoluble cytoplasmic fraction) was washed by resuspension in homogenization buffer followed by centrifugation at 100 000 *g*, 4 °C for 15 min and was then resuspended in homogenization buffer. All three fractions were acetone precipitated and resuspended in an equal amount of sample buffer for immunoblot analysis.

### Proteasome inhibition

The proteasome inhibitor MG132 (Merck) was diluted in DMSO. Cells were treated with MG132 at a final concentration of 10 μM for 5 h. Cells treated only with DMSO served as controls.

### Immunoblot analysis

Cells were harvested by scraping in cold PBS, centrifuged at 900 *g* for 5 min at room temperature, and lysed in 50 mm Tris/HCl pH 7.5, 200 mm NaCl, 5 mm EDTA, 0.1% NP40, 0.5% Tween 20, and 1 mm PMSF supplemented with Complete protease inhibitors (Roche Diagnostics, Mannheim, Germany). Proteins were separated by SDS/PAGE using NuPAGE 3–8% Tris‐Acetate or Bolt 4–12% Bis‐Tris mini‐gels (ThermoFisher Scientific) and transferred to nitrocellulose membranes. After blocking, membranes were incubated overnight at 4 °C with anti‐ZNF469 antibodies diluted 1:1000, followed by HRP‐linked anti‐rabbit IgG antibodies as described previously [27]. A 20–220 kDa (Magic Mark; ThermoFisher Scientific) and a 30–460 kDa protein standard (HiMark; ThermoFisher Scientific) were used as molecular weight markers. After incubation with the Super Signal West Pico Substrate (Thermo Fisher Scientific), protein bands were visualized using a ChemiDoc Touch Imaging System (Bio‐Rad). GAPDH served as a loading control.

### Immunofluorescence analysis

Double labeling immunofluorescence analysis was performed according to Sannerud and co‐workers with minor modifications [28]. Cells were grown on poly‐L‐lysine coated coverslips, rinsed in cold PBS, fixed with 4% paraformaldehyde in 0.1 M phosphate buffer pH 7.5 for 30 min, and kept overnight at 4 °C in washing buffer. After permeabilization (5 min) and treatment with normal goat serum (30 min), the cells were incubated with primary antibodies against ZNF469, followed by antibodies against various subcellular marker proteins. The coverslips were then washed and incubated with secondary antibodies (antibody dilutions have been reported previously [26]). Each incubation and washing step was carried out at 4 °C for a minimum of 12 h. The coverslips were mounted using ProLong Gold with DAPI (ThermoFisher Scientific). Negative controls included normal rabbit serum instead of ZNF469 antibodies. The specimens were examined using Leica SP5 or SP8 confocal laser scanning microscopes (Leica Microsystems, Wetzlar, Germany). The Lightning deconvolution software was used to enhance image resolution.

### Live cell imaging

HEK293 cells stably expressing ZNF469 with a C‐terminal GFP tag were seeded in 35 mm culture dishes (no. 1.5 uncoated coverslip, 14 mm glass diameter) (#P35G‐1.5‐14‐C; MatTek) for live cell imaging. A Nikon TE2000 widefield microscope equipped with a 20× ELWD Plan Fluor objective (NA 0.45) and a DS‐QI1MC‐U2 camera, laser line 488 nm, was used for image capture under temperature and CO_2_ control (37 °C and 5% CO_2_). Imaging was performed overnight with images captured every 15 min.

### Response to stress

Wild‐type U2OS, U2OS transfected with ZNF469‐GFP, and U2OS ∆∆G3BP1/2 cells transduced with mCherry‐G3BP1 were incubated with either 200 μM sodium arsenite (# 41533; Alfa Aesar/ThermoFisher Scientific) in DMEM, 20 μM clotrimazole (# C6019, Sigma/Merck) in Opti‐MEM, 100 μM MG132 (# 474790; Sigma/Merck) in DMEM, or 0.4 M D‐Sorbitol (# S6021; Sigma/Merck) in DMEM for 60 min prior to fixation and permeabilization in 100% methanol for 5 min. Stress granules were identified via the mCherry‐G3BP1 construct (excitation wavelength 587 nm, emission wavelength 610 nm) or through incubation with an antibody raised against G3BP1, as described above.

### Fluorescence *in situ* hybridization

FISH was performed according to (10.21769/BioProtoc.999) [29]. Briefly, immunolabeled cells were re‐fixed in 4% paraformaldehyde with 0.1% Triton‐X100 for 10 min at room temperature. Cells were incubated with 0.5 μM 5′‐Cy5‐oligo d(T)30 against polyadenylated RNA (Integrated DNA Technologies, IA, USA) in hybridization buffer (70% formamide, 10 mm Tris/HCL [pH 7.4]) at 80 °C for 5 min and then for 120 min in a humidified chamber at room temperature. Blue pseudocolor was used to visualize the Cy5 signal.

### Reproducibility

The number of technical and biological replicates is indicated in the figure legends. In the supplementary figures, all immunoblots and Fig. S5 represent examples from three biological replicates, while the remaining immunofluorescence images are examples from experiments performed in three technical replicates.

## Results

### *In silico* predictions

Apart from the zinc finger domains, the most prominent feature of ZNF469 is the presence of disordered regions. Approximately 74% of the protein is made up of disordered regions according to MobiDB. A total of 36 low‐complexity domains were present in the disordered regions according to the SMART database. Additional analysis with different predictors included in the D2P2 and RIDAO databases confirmed the presence of extensive disordered regions. The D2P2 analysis also revealed a potential methylation site and several putative phosphorylation sites. Molecular recognition features (MoRFs) represent functional regions that may undergo a transition from disorder to order by binding to their interaction partners. MoRFs were seen along the entire length of ZNF469. Five (one overlapping) classical monopartite nuclear localization signals were recognized by cNLS Mapper. No nuclear export signals were detected using the LocNES tool. An overview of the *in silico* predictions is shown in Fig. S1.

### Endogenous expression of ZNF469

#### Quantitative PCR

**Fig. 1:**
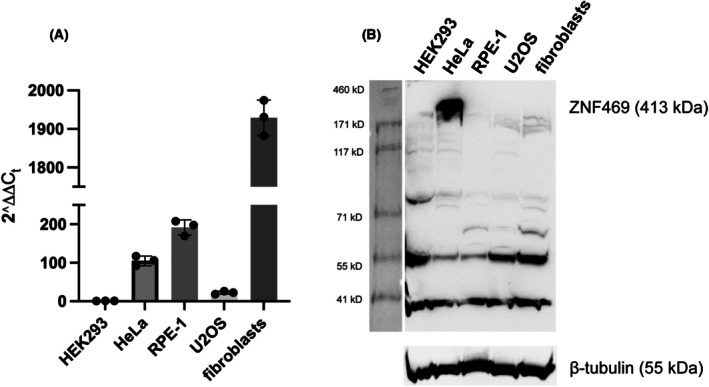
ZNF469 RNA and protein expression levels were measured by quantitative PCR (A) and immunoblot analysis (B) of ZNF469 in different cell lines. (A) After amplification with TaqMan probes, quantitative PCR results were analyzed using the ∆∆Ct method, normalizing the ZNF469 levels to the reference gene HPRT1. Results were obtained from a total of 9 replicates for each cell line. Error bars indicate SD. (B) The HiMark molecular weight standard is shown to the left. Representative blots from three biological replicates are presented.

For comparison with the data presented by the Human Protein Atlas, we performed quantitative PCR analysis of the ZNF469 RNA expression level in HEK293, Hela, U2OS, and RPE1 cells as well as primary human skin fibroblasts. Highest levels were detected in primary fibroblasts, while low levels were seen in HEK293 cells. The relative levels of ZNF469 mRNA is shown in Fig. 1A. Ct‐values are presented in Fig. S2A.

#### Anti‐ZNF469 antibody and immunoblot analysis

**Fig. 2:**
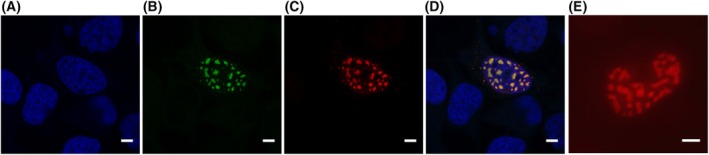
(A–D) HEK293 cells transfected with plasmids expressing a ZNF469‐AcGFP fusion protein (green) were labeled with antibodies against ZNF469 (red). Nuclei are shown in blue. An overlay with double labeled nuclear speckles in shown in yellow. (E) HEK293 cells transfected with a plasmid expressing ZNF469 without the AcGFP tag incubated with antibodies against ZNF469 (red). The experiments were performed in three biological replicates with similar results. Scale bar: 5 μm.

We first examined the ZNF469 antibody in transfected cells overexpressing a ZNF469‐AcGFP fusion protein. A speckled nuclear fluorescence signal was observed in transfected cells. There was complete overlap of the green fluorescent protein signal and the signal produced by the antibody (Fig. 2) showing that the antibodies recognized the ZNF469 protein.

In immunoblot analysis of whole cell lysates, the ZNF469 antibody recognized a double band corresponding to protein(s) with a MW of approximately 400 kDa in HeLa cells (Fig. 1B). In a separate experiment, a ~400 kDa protein band was also seen in whole cell lysates from primary human fibroblasts (Fig. S3G). Several smaller sized protein bands were also seen. It is not clear if these are fragments or degradation products of ZNF469 or if there is cross‐reaction with other proteins.

#### Subcellular fractionation

**Fig. 3:**
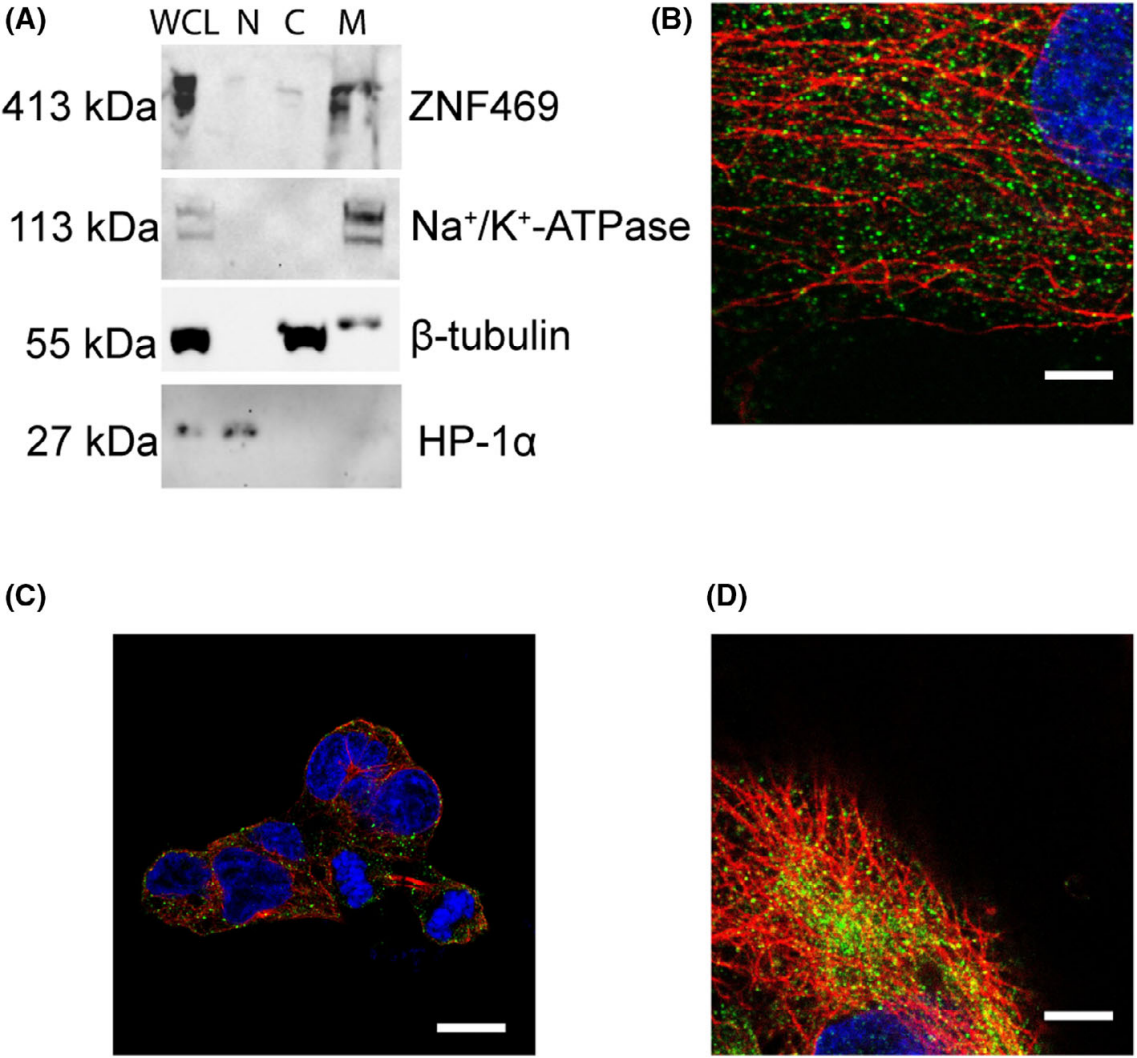
Intracellular localization of ZNF469 in interphase cells. (A) Subcellular fractionation of HeLa cells. Immunoblot showing endogenously expressed proteins detected by anti‐ZNF469 antibodies. C, soluble cytosolic fraction; M, insoluble cytosolic fraction; N, nuclear fraction; WCL, whole cell lysates. HP1α was used as marker for the nuclear fraction while β‐tubulin and Na^+^/K^+^‐ATPase were used as markers for the soluble and insoluble cytosolic fraction, respectively. The blots represent examples from three biological replicates. (B–D) Immunofluorescence analysis using anti‐ZNF469 antibodies (green) and antibodies against β‐tubulin (red) in primary fibroblasts from a healthy individual (B), HEK293 cells (C), and HeLa cells (D). Similar results were obtained in three biological replicates. Scale bar: 5 μm.

Since the ~400 kDa protein band was most prominent in HeLa cells, we subjected HeLa cells to subcellular fractionation. The cells were lysed and separated into a nuclear and two cytoplasmic fractions. Antibodies against Na^+^/K^+^‐ATP‐ase and β‐tubulin were used as markers for the insoluble (“membrane”) and the soluble (“cytosol”) cytoplasmic fractions, respectively, while anti‐HP1α antibodies were used for the nuclear fraction. Immunoblot analysis revealed that the proteins recognized by the anti‐ZNF469 antibodies were present mainly in the insoluble (“membrane”) fraction of the cytoplasm (Fig. 3A, complete blots in Fig. S2). This included the ~400 kDa protein anticipated to represent full‐length ZNF469.

As described below, we observed punctate staining of the cytoplasm in HEK293 cells (Fig. 3C). This was surprising since HEK293 cells showed low levels of ZNF469 mRNA and undetectable levels of the ~400 kDa protein in immunoblot analysis of whole cell lysates. After subcellular fractionation, however, a ~400 kDa protein was detected in the insoluble fraction of the cytoplasm (Fig. S2).

#### Immunofluorescence analysis

Immunofluorescence analysis revealed punctate cytoplasmic staining in interphase cells (Fig. 3B–D; Figs S3, S7, S8). In HeLa cells, some of the staining was localized along microtubules. In double labeling experiments of primary fibroblasts, the pattern of ZNF469 antibody staining did not correspond to any of the major cytoplasmic organelles including endoplasmic reticulum (ER) (SPC‐25), Golgi (GM130), inner (ATPIF1) or outer (TOMM20) mitochondrial membrane, and lysosomes (LAMP1), although occasionally the staining would overlap (Fig. S3). A weak, punctate nuclear staining was seen in most cell lines except for HEK293 cells (Fig. 3, Figs S3, S7, S8). In stressed U2OS cells, a weak homogeneous nuclear staining was seen (Figs S7, S8).

**Fig. 4:**
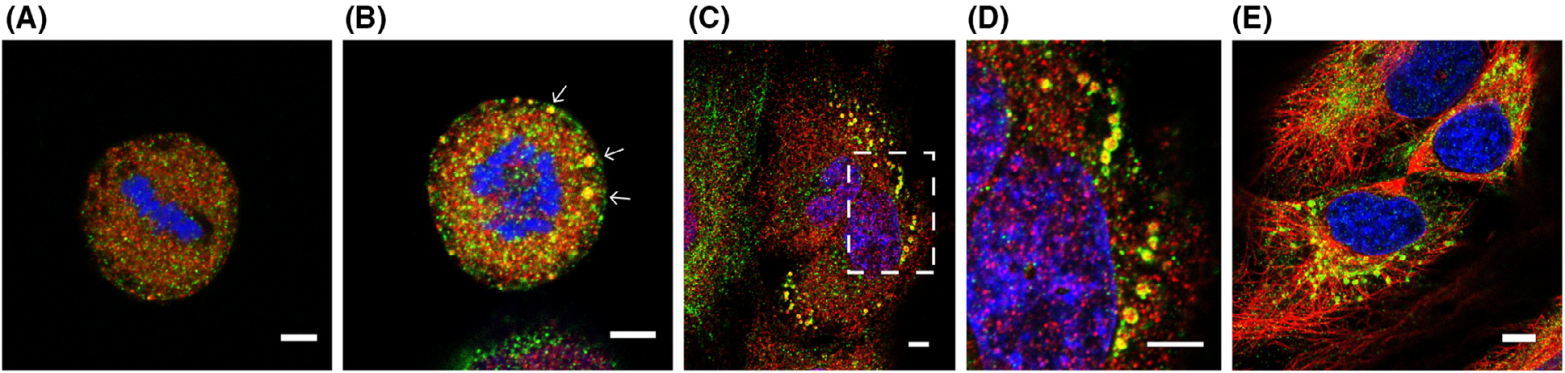
Double labeling immunofluorescence analysis of dividing HeLa cells (A–E). HeLa cells were incubated with antibodies against ZNF469 (green) and 19 S proteasomes (red) (A–D) or β‐tubulin (red) (E). (A and B) Metaphase. (C–E) Telophase (abscission). (D) Enlarged image from (C). Arrows in (B) and yellow‐white staining in (C and D) indicate overlapping staining for ZNF469 and 19 S proteasomes. Example images from three biological replicates are shown. Scale bar: 5 μm.

The staining of the anti‐ZNF469 antibodies during mitosis was examined in HeLa cells and primary fibroblasts. In fibroblasts, the staining was mainly punctate (Fig. S4), while in HeLa cells, larger foci also appeared (Fig. 4). In double labelling experiments with antibodies against 19S proteasomes, complete overlap was seen in some of these foci, while in others, the ZNF469 staining surrounded the 19S proteasome staining in a doughnut‐like shape. The foci appeared early in mitosis, persisted through the final stages of cytokinesis, and were seen in the cytoplasm of the daughter cells shortly after the nucleus had reappeared (Fig. 4). Some overlapping staining with proteasomes was seen of the puncta in primary fibroblasts as well, but the characteristic doughnut‐shaped foci were not observed (Fig. S4). In some primary fibroblasts (<5%), doughnut‐shaped dots appeared in the cytoplasm during interphase, as shown in Fig. S3F. Incubation with the proteasome inhibitor MG132 resulted in an increase in the amount of the ~400 kDa protein recognized by the anti‐ZNF469 antibodies (Fig. S3G).

### Transfection with ZNF469

**Fig. 5:**
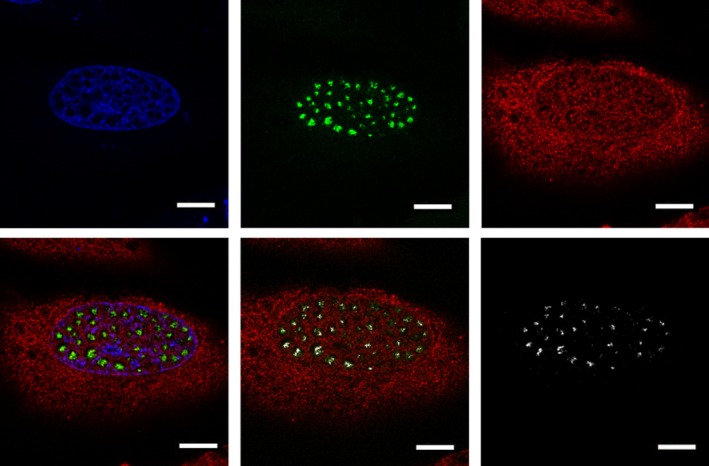
HeLa cells transfected with plasmids expressing the ZNF469‐AcGFP fusion protein (green) and labeled with antibodies against 19 S proteasome (red). Nuclei are shown in blue. White signal indicates overlapping staining of ZNF469‐AcGFP and 19 S proteasomes. The experiment was repeated twice, each in three technical replicates. Scale bar: 5 μm.

**Fig. 6:**
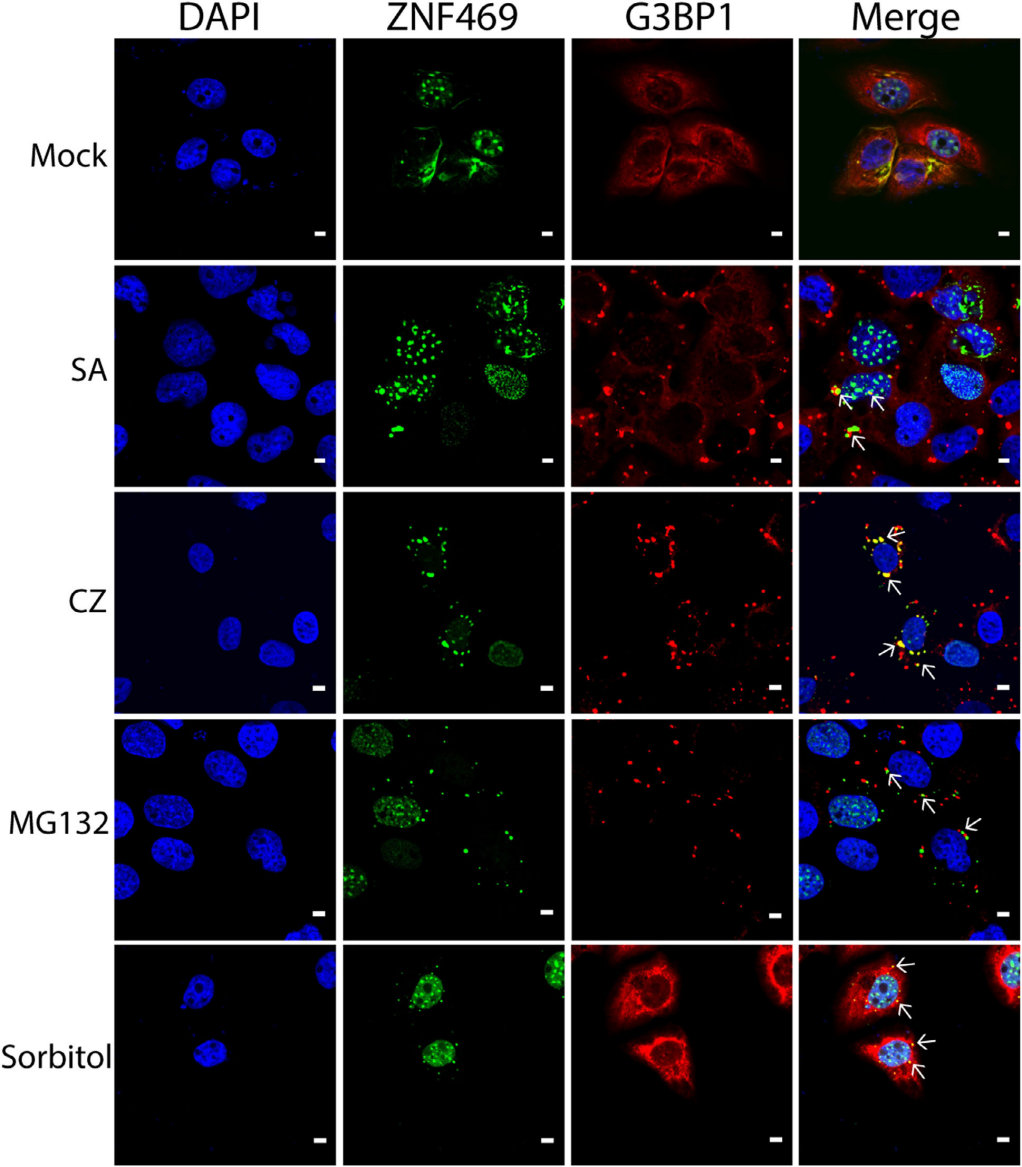
Expression of ZNF469 during stress granule formation in wt U2OS cells transfected with ZNF469‐AcGFP (green). Anti‐G3BP1 antibodies were used to detect the stress granule marker G3BP1 (red). Stress modes included sodium arsenite (SA), clotrimazole (CZ), MG132, and sorbitol. Arrows indicate stress granules with overlapping staining of ZNF469 and G3BP1. All experiments were conducted in three technical replicates. Scale bar: 5 μm.

We transfected HeLa, HEK293, GM847, and U2OS cells with the plasmid expressing the ZNF469‐AcGFP fusion protein. HEK293 cells were used since they are easy to transfect, GM847 cells because they express PML bodies, and U2OS cells for stress granule experiments. A strong speckled nuclear fluorescence signal appeared in interphase cells (Figs 2, 5, 6; Figs S5A, S6). In some cells, foci were also seen in the cytoplasm. As a control for the effect of the GFP tag, we transfected HEK293 cells with a plasmid expressing ZNF469 without the GFP tag. In immunofluorescence analysis, the ZNF469 antibodies produced a speckled nuclear staining as seen in cells transfected with *ZNF469‐AcGFP*, indicating that the GFP tag did not affect the localization of ZNF469 (Fig. 2).

In mitotic cells, staining of the midbody was a prominent feature (Fig. S5D and Video S1). In addition, small foci were seen in HeLa cells, most prominent in abscission, where they were detected opposite to the midbody (Fig. S5D). In GM847 cells, GFP staining of the mitotic spindle was observed from late metaphase to telophase (Fig. S5). We also observed staining of cytoplasmic microtubules in the interphase of GM847 and U2OS cells (Fig. S5A; Fig. 6).

Double labeling immunofluorescence experiments with antibodies against proteins known to be present in nuclear speckles including sc35, U1snRNP, PML, SUMO, coilin, HP1α, and TRF‐2 were performed in GM847 cells. We did not observe any overlapping staining with ZNF469‐AcGFP (Fig. S6).

Antibodies against proteasomes, however, reacted with the nuclear foci as shown in HeLa cells (Fig. 5). By using the Lightning deconvolution module, punctate overlapping staining was seen in parts of the nuclear foci.

### Live cell imaging

To further characterize the nuclear foci produced by ZNF469 in transfected cells, we performed live cell imaging. Since *in silico* analysis had revealed the presence of large, disordered regions with multiple low‐complexity domains in ZNF469, we hypothesized that the foci could represent biomolecular condensates. In stably transfected HEK293 cells expressing GFP‐tagged ZNF469, nuclear foci were seen resembling the foci observed in transiently transfected cells. The foci were dynamic in nature as their shape and position changed during the recording (Video S2). We observed splitting or fusion of some foci, indicating that they had liquid‐like properties.

### Formation of stress granules

Stress granules are well‐characterized examples of biomolecular condensates [30]. We therefore examined ZNF469 during stress granule formation in U2OS cells using G3BP1 and oligo d(T) as markers. In untreated, native cells, punctate staining of anti‐ZNF469 antibodies was seen in the cytoplasm and in some nuclei. After treatment with sodium arsenite or clotrimazole, we observed overlapping staining of antibodies against ZNF469 and G3BP1 in a few granules, suggesting that proteins recognized by the anti‐ZNF469 antibodies could participate in the formation of stress granules (Fig. S7). In untreated transfected cells, stress granules appeared that did not show any overlapping staining with ZNF469, suggesting that the transfection itself represented a stressful event for the cells. Aggregates of ZNF469 and stress granules were frequently located next to each other (Fig. 6). After sorbitol treatment, some stress granules appeared that showed overlapping staining of G3BP1 and ZNF469‐AcGFP (Fig. 6). In ΔΔG3BP1/2 U2OS cells transduced with mCherry‐G3BP1, a prominent reaction of anti‐ZNF469 antibodies with stress granules was seen in all stress modes tested (Fig. S8).

## Discussion

In this report, we present data showing that anti‐ZNF469 antibodies recognize a protein with the appropriate molecular weight that is localized in the cytoplasm. In contrast, when overexpressed in transfected cells, ZNF469 is seen mainly in nuclear foci. These foci split and fuse during live cell imaging, suggesting that they could represent biomolecular condensates.

The anti‐ZNF469 antibody reacted only to a minor extent with the nuclei, which is in contrast to the localization reported in the Human Protein Atlas (https://www.proteinatlas.org/search/ZNF469). We observed a cytoplasmic localization both in subcellular fractionation experiments and in immunofluorescence analysis. The nuclear localization reported in the Human Protein Atlas was shown for U2OS cells. The cells where we observed the most prominent nuclear reaction were also U2OS cells. It is possible that the extent of nuclear localization may vary between cell lines. In immunoblot analysis, we observed in addition to the ~400 kDa protein several other protein bands. They could represent degradation products or alternatively proteins cross‐reacting with the anti‐ZNF469 antibodies, which could bias the immunofluorescence analysis. In HEK293 cells, unexpected results were seen in that a ~400 kDa protein appeared in the insoluble fraction of the cytoplasm that was not detected in immunoblot analysis of whole cell lysates. To some extent, this could be explained by the relatively small volume of the cytoplasm relative to the nucleus in HEK293 cells compared to HeLa cells.

ZNF469 has been shown to play a role in regulating the transcription of ECM proteins [1, 4, 12]. We identified several nuclear localization signals in ZNF469 and therefore expected ZNF469 to be localized to the nucleus. Many transcription factors, however, reside in the cytoplasm and are transported to the nucleus only in response to specific stimuli [31, 32]. In the cytoplasm, they can be associated with membranes like the plasma membrane and the ER, or they can be present in macromolecular complexes in the cytosol [33]. Release for transport into the nucleus may involve proteolytic cleavage of the transcription factor from the membrane [34] or degradation of inhibitory factors that otherwise prevent nuclear transport [33].

In transfected cells, we observed nuclear localization of ZNF469 in multiple nuclear foci, as previously reported by Ariyachet *et al*. [12]. Both overexpression and tagging may result in mis‐localization of proteins, among others, because of overloading of the normal cellular localization machinery [35]. In cells overexpressing ZNF469 without a tag, similar nuclear foci appeared, as seen in cells overexpressing ZNF469 with a GFP tag, indicating that the tag did not influence the localization of ZNF469. The nuclear localization in transfected cells could indicate that when overexpressed, ZNF469 may titrate out inhibitory factors that otherwise prevent nuclear localization.

Overexpression may also cause erroneous formation of protein complexes by exceeding the cell's capacity for degradation, folding, or intracellular transport [36, 37]. Overexpressed or misfolded proteins can be sequestered into compartments including aggresomes [38], nucleoli [39], and stress granules [40, 41]. In some cell types, dispersed aggregates may appear instead of aggresomes [42]. Recently, proteasome‐containing foci have been shown to form in the nuclei of cells both at basal conditions and in cells exposed to various forms of stress [43, 44]. These foci are active sites of proteolysis and represent biomolecular condensates that are not co‐localized with known nuclear membrane‐less organelles [43, 44]. The nuclear foci seen in cells overexpressing ZNF469 showed overlapping staining with proteasomes and therefore resemble such proteasome‐containing foci. Live cell imaging of the ZNF469 nuclear foci revealed that they changed shape and position and would occasionally split or fuse, which is characteristic of the liquid‐like property of biomolecular condensates [45].

*In silico* analysis showed that ZNF469 contains large intrinsically disordered regions with multiple low complexity domains [46]. Although not essential [21], such regions may contribute to liquid–liquid phase separation of the protein [47, 48]. Stress granules are typical examples of biomolecular condensates [49, 50]. While ZNF469 has not been detected in screens of stress granules [51], we found that the anti‐ZNF469 antibodies recognized proteins that participated in stress granule formation, in particular in ΔΔG3BP1/2 U2OS cells transduced with mCherry‐G3BP1, supporting that they may form biomolecular condensates. Why the co‐localization appeared mainly in these cells is not clear. It could be related to the concentration of key constituents for stress granule formation, like G3BP1 [21].

A consistent feature seen in transfected cells was the co‐localization with the mitotic spindle, particularly at late stages of mitosis. Several zinc finger proteins are involved in spindle assembly and function, including chromosome alignment maintaining phosphoprotein (CAMP) (ZNF828) [52] and BuGZ (ZNF207) [53]. However, ZNF469 has not been identified in proteome analysis of purified mitotic spindles [54] or midbodies [55]. Microtubules can be a surface for biomolecular condensate formation, resulting in “wetting” of the microtubules [56]. Whether the staining of the midbody reflects a functional feature of ZNF469 or is just a consequence of ZNF469 overexpression remains to be determined.

The potential for ZNF469 to participate in the formation of biomolecular condensates could be relevant for cells expressing endogenous levels of ZNF469. In HeLa cells, foci where ZNF469 surrounded proteasomes were seen. These foci could represent multiphase droplets [57] with the proteasomes in the core and ZNF469 in the shell. Fu and co‐workers described proteasomal foci where the ubiquitinated substrate was localized in the center and the proteasomes in the periphery [44]. A combined aggregate and condensate could also be present since in many biomolecular condensates with a complex structure not all constituents are present in a liquid form [50, 51]. In a recent paper, Bao and co‐workers observed that loss of ZNF469 resulted in increased expression of proteasomal proteins in zebrafish [11]. The relationship between ZNF469 and proteasomes needs to be explored further.

### Conclusion

Anti‐ZNF469 antibodies recognized a ~ 400 kDa protein that was localized mainly in the cytoplasm. When overexpressed, nuclear localization in large foci was seen. These foci resemble biomolecular condensates. ZNF469 contains large, disordered regions which may explain its tendency to aggregate and possibly form biomolecular condensates. Many transcription factors reside in the cytoplasm and are transported to the nucleus only when needed. A cytoplasmic localization of ZNF469 is therefore compatible with its proposed role as a transcriptional regulator. Due to the discrepancy in the localization of endogenous and overexpressed ZNF469, care must be taken when interpreting the data.

## Conflicts of interest

A.E.C. Mellgren, None; I. Cristea, None; T. Stevenson, None; E. Spriet, None; P.M. Knappskog, None; S.O. Bøe, None; H. Kranz, None; S.N. Grellscheid, None; E. Rødahl, None.

## Peer review

The peer review history for this article is available at https://www.webofscience.com/api/gateway/wos/peer‐review/10.1002/2211‐5463.70034.

## Author contributions

AECM, TS, SNG, and ER conceived the study; SNG and ER supervised the study. AECM, TS, ES, IC, and ER conducted experiments. HK provided ZNF469 plasmids. All authors analyzed results. AECM, IC, and ER wrote the manuscript. All authors reviewed and approved the manuscript.

## Supporting information


**Fig. S1** Schematic representation of the ZNF469 protein with predictions of disordered regions.

**Fig. S2** (A) Average Ct values from the quantitative PCR analysis shown in Fig. 1. (B) Full blots of the subcellular fractionation shown in Fig. 3. (C) Subcellular fractionation of HEK293 cells.

**Fig. S3** (A–F) Immunofluorescence analysis of fibroblasts using antibodies against ZNF469 and proteins associated with cytoplasmic organelles. (G) Immunoblot analysis of fibroblast extracts without and after treatment with MG132.

**Fig. S4** Immunofluorescence analysis of fibroblasts in metaphase using antibodies against ZNF469 and 19S proteasomes.

**Fig. S5** (A–C) Transfected GM847 cells expressing ZNF469‐AcGFP fusion protein labeled with antibodies against acetylated tubulin. (D) Transfected HeLa cells expressing ZNF469‐AcGFP fusion protein during abscission.

**Fig. S6** Immunofluorescence analysis of GM847 cells transfected with ZNF469‐AcGFP labeled with antibodies against nuclear proteins.

**Fig. S7** Expression of ZNF469 during stress granule formation in wt U2OS cells using anti‐ZNF469 antibodies.

**Fig. S8** Expression of ZNF469 during stress granule formation in ΔΔG3BP1/2 U2OS cells transfected with mCherry‐G3BP1.

**Video S1** Live cell imaging of stably transfected HEK293 cells expressing ZNF469‐AcGFP fusion protein. During abscission, staining of the midbody is seen.

**Video S2** Live cell imaging of stably transfected HEK293 cells expressing ZNF469‐AcGFP fusion protein. In interphase, the nuclear foci vary in size and some foci split or fuse during the recording.

## Data Availability

The data that support the findings of this study are available in Figs [Link], [Link], [Link], [Link], [Link], [Link], [Link], [Link], Videos S1 and S2.

## References

[1] Rohrbach M , Spencer HL , Porter LF , Burkitt‐Wright EM , Bürer C , Janecke A , Bakshi M , Sillence D , Al‐Hussain H , Baumgartner M *et al*. (2013) ZNF469 frequently mutated in the brittle cornea syndrome (BCS) is a single exon gene possibly regulating the expression of several extracellular matrix components. Mol Genet Metab 109, 289–295. 23680354 10.1016/j.ymgme.2013.04.014 PMC3925994

[2] Abu A , Frydman M , Marek D , Pras E , Nir U , Reznik‐Wolf H and Pras E (2008) Deleterious mutations in the zinc‐finger 469 gene cause brittle cornea syndrome. Am J Hum Genet 82, 1217–1222. 18452888 10.1016/j.ajhg.2008.04.001 PMC2427192

[3] Christensen AE , Knappskog PM , Midtbø M , Gjesdal CG , Mengel‐From J , Morling N , Rødahl E and Boman H (2010) Brittle cornea syndrome associated with a missense mutation in the zinc‐finger 469 gene. Invest Ophthalmol Vis Sci 51, 47–52. 19661234 10.1167/iovs.09-4251

[4] Burkitt Wright EMM , Spencer HL , Daly SB , Manson FDC , Zeef LAH , Urquhart J , Zoppi N , Bonshek R , Tosounidis I , Mohan M *et al*. (2011) Mutations in PRDM5 in brittle cornea syndrome identify a pathway regulating extracellular matrix development and maintenance. Am J Hum Genet 88, 767–777. 21664999 10.1016/j.ajhg.2011.05.007 PMC3113239

[5] Stanton CM , Findlay AS , Drake C , Mustafa MZ , Gautier P , McKie L , Jackson IJ and Vitart V (2021) A mouse model of brittle cornea syndrome caused by mutation in Zfp469. Dis Model Mech 14, dmm049175. 34368841 10.1242/dmm.049175 PMC8476817

[6] Lu Y , Dimasi DP , Hysi PG , Hewitt AW , Burdon KP , Toh T , Ruddle JB , Li YJ , Mitchell P , Healey PR *et al*. (2010) Common genetic variants near the brittle cornea syndrome locus ZNF469 influence the blinding disease risk factor central corneal thickness. PLoS Genet 6, e1000947. 20485516 10.1371/journal.pgen.1000947 PMC2869325

[7] Gao X , Gauderman WJ , Liu Y , Marjoram P , Torres M , Haritunians T , Kuo JZ , Chen YD , Allingham RR , Hauser MA *et al*. (2013) A genome‐wide association study of central corneal thickness in Latinos. Invest Ophthalmol Vis Sci 54, 2435–2443. 23493294 10.1167/iovs.13-11692 PMC3621577

[8] Rong SS , Ma STU , Yu XT , Ma L , Chu WK , Chan TCY , Wang YM , Young AL , Pang CP , Jhanji V *et al*. (2017) Genetic associations for keratoconus: a systematic review and meta‐analysis. Sci Rep 7, 4620. 28676647 10.1038/s41598-017-04393-2 PMC5496893

[9] Fransen E , Valgaeren H , Janssens K , Sommen M , De Ridder R , Vandeweyer G , Bisceglia L , Soler V , Hoischen A , Mortier G *et al*. (2021) Resequencing of candidate genes for Keratoconus reveals a role for Ehlers‐Danlos syndrome genes. Eur J Hum Genet 29, 1745–1755. 33737726 10.1038/s41431-021-00849-2 PMC8633318

[10] Lucas SEM , Zhou T , Blackburn NB , Mills RA , Ellis J , Leo P , Souzeau E , Ridge B , Charlesworth JC , Brown MA *et al*. (2017) Rare, potentially pathogenic variants in ZNF469 are not enriched in Keratoconus in a large Australian cohort of European descent. Invest Ophthalmol Vis Sci 58, 6248–6256. 29228253 10.1167/iovs.17-22417

[11] Bao J , Yu X , Ping X , Shentu X and Zou J (2023) Znf469 plays a critical role in regulating synthesis of ECM: a zebrafish model of brittle cornea syndrome. Invest Ophthalmol Vis Sci 64, 29. 10.1167/iovs.64.5.29 PMC10233312 37256609

[12] Ariyachet C , Nokkeaw A , Boonkaew B and Tangkijvanich P (2024) ZNF469 is a profibrotic regulator of extracellular matrix in hepatic stellate cells. J Cell Biochem 125, e30578. 38704698 10.1002/jcb.30578

[13] Paysan‐Lafosse T , Blum M , Chuguransky S , Grego T , Pinto BL , Salazar GA , Bileschi ML , Bork P , Bridge A , Colwell L *et al*. (2023) InterPro in 2022. Nucleic Acids Res 51, D418–D427. 36350672 10.1093/nar/gkac993 PMC9825450

[14] Letunic I , Khedkar S and Bork P (2021) SMART: recent updates, new developments and status in 2020. Nucleic Acids Res 49, D458–D460. 33104802 10.1093/nar/gkaa937 PMC7778883

[15] Piovesan D , Del Conte A , Clementel D , Monzon AM , Bevilacqua M , Aspromonte MC , Iserte JA , Orti FE , Marino‐Buslje C and Tosatto SCE (2023) MobiDB: 10 years of intrinsically disordered proteins. Nucleic Acids Res 51(D1), D438–D444. 36416266 10.1093/nar/gkac1065 PMC9825420

[16] Oates ME , Romero P , Ishida T , Ghalwash M , Mizianty MJ , Xue B , Dosztanyi Z , Uversky VN , Obradovic Z , Kurgan L *et al*. (2013) D(2)P(2): database of disordered protein predictions. Nucleic Acids Res 41, D508–D516. 23203878 10.1093/nar/gks1226 PMC3531159

[17] Dayhoff GW 2nd and Uversky VN (2022) Rapid prediction and analysis of protein intrinsic disorder. Protein Sci 31, e4496. 36334049 10.1002/pro.4496 PMC9679974

[18] Kosugi S , Hasebe M , Tomita M and Yanagawa H (2009) Systematic identification of cell cycle‐dependent yeast nucleocytoplasmic shuttling proteins by prediction of composite motifs. Proc Natl Acad Sci U S A 106, 10171–10176. 19520826 10.1073/pnas.0900604106 PMC2695404

[19] Xu D , Marquis K , Pei J , Fu SC , Cagatay T , Grishin NV and Chook YM (2015) LocNES: a computational tool for locating classical NESs in CRM1 cargo proteins. Bioinformatics 31, 1357–1365. 25515756 10.1093/bioinformatics/btu826 PMC4410651

[20] Kedersha N , Panas MD , Achorn CA , Lyons S , Tisdale S , Hickman T , Thomas M , Lieberman J , McInerney GM , Ivanov P *et al*. (2016) G3BP‐Caprin1‐USP10 complexes mediate stress granule condensation and associate with 40S subunits. J Cell Biol 212, 845–860. 27022092 10.1083/jcb.201508028 PMC4810302

[21] Sanders DW , Kedersha N , Lee DSW , Strom AR , Drake V , Riback JA , Bracha D , Eeftens JM , Iwanicki A , Wang A *et al*. (2020) Competing protein‐RNA interaction networks control multiphase intracellular organization. Cell 181, 306–324. 32302570 10.1016/j.cell.2020.03.050 PMC7816278

[22] Pereira‐Smith OM and Smith JR (1981) Expression of SV40 T antigen in finite life‐span hybrids of normal and SV40‐transformed fibroblasts. Somatic Cell Genet 7, 411–421. 6269237 10.1007/BF01542986

[23] Croce CM , Girardi AJ and Koprowski H (1973) Assignment of the T‐antigen gene of simian virus 40 to human chromosome C‐7. Proc Natl Acad Sci U S A 70, 3617–3620. 4357883 10.1073/pnas.70.12.3617 PMC427292

[24] Bredrup C , Stokowy T , McGaughran J , Lee S , Sapkota D , Cristea I , Xu L , Tveit KS , Høvding G , Steen VM *et al*. (2019) A tyrosine kinase‐activating variant Asn666Ser in PDGFRB causes a progeria‐like condition in the severe end of Penttinen syndrome. Eur J Hum Genet 27, 574–581. 30573803 10.1038/s41431-018-0323-z PMC6460636

[25] Dai C , Cao Z , Wu Y , Yi H , Jiang D and Li W (2007) Improved fusion protein expression of EGFP via the mutation of both Kozak and the initial ATG codon. Cell Mol Biol Lett 12, 362–369. 17318296 10.2478/s11658-007-0008-z PMC6275746

[26] Cristea I , Bruland O , Aukrust I , Rødahl E and Bredrup C (2021) Pellino‐2 in nonimmune cells: novel interaction partners and intracellular localization. FEBS Lett 595, 2909–2921. 34674267 10.1002/1873-3468.14212

[27] Mellgren AE , Bruland O , Vedeler A , Saraste J , Schönheit J , Bredrup C , Knappskog PM and Rødahl E (2015) Development of congenital stromal corneal dystrophy is dependent on export and extracellular deposition of truncated decorin. Invest Ophthalmol Vis Sci 56, 2909–2915. 26029887 10.1167/iovs.14-16014

[28] Sannerud R , Marie M , Hansen BB and Saraste J (2008) Use of polarized PC12 cells to monitor protein localization in the early biosynthetic pathway. Methods Mol Biol 457, 253–265. 19066033 10.1007/978-1-59745-261-8_19

[29] Fumagalli M , Rossiello F , Clerici M , Barozzi S , Cittaro D , Kaplunov JM , Bucci G , Dobreva M , Matti V , Beausejour CM *et al*. (2012) Telomeric DNA damage is irreparable and causes persistent DNA‐damage‐response activation. Nat Cell Biol 14, 355–365. 22426077 10.1038/ncb2466 PMC3717580

[30] Protter DSW and Parker R (2016) Principles and properties of stress granules. Trends Cell Biol 26, 668–679. 27289443 10.1016/j.tcb.2016.05.004 PMC4993645

[31] Xu L and Massague J (2004) Nucleocytoplasmic shuttling of signal transducers. Nat Rev Mol Cell Biol 5, 209–219. 14991001 10.1038/nrm1331

[32] Sweeney K and McClean MN (2023) Transcription factor localization dynamics and DNA binding drive distinct promoter interpretations. Cell Rep 42, 112426. 37087734 10.1016/j.celrep.2023.112426 PMC10292158

[33] Oeckinghaus A and Ghosh S (2009) The NF‐kappaB family of transcription factors and its regulation. Cold Spring Harb Perspect Biol 1, a000034. 20066092 10.1101/cshperspect.a000034 PMC2773619

[34] Liu Y , Li P , Fan L and Wu M (2018) The nuclear transportation routes of membrane‐bound transcription factors. Cell Commun Signal 16, 12. 29615051 10.1186/s12964-018-0224-3 PMC5883603

[35] Kintaka R , Makanae K and Moriya H (2016) Cellular growth defects triggered by an overload of protein localization processes. Sci Rep 6, 31774. 27538565 10.1038/srep31774 PMC4990933

[36] Gibson TJ , Seiler M and Veitia RA (2013) The transience of transient overexpression. Nat Methods 10, 715–721. 23900254 10.1038/nmeth.2534

[37] Moriya H (2015) Quantitative nature of overexpression experiments. Mol Biol Cell 26, 3932–3939. 26543202 10.1091/mbc.E15-07-0512 PMC4710226

[38] Kopito RR (2000) Aggresomes, inclusion bodies and protein aggregation. Trends Cell Biol 10, 524–530. 11121744 10.1016/s0962-8924(00)01852-3

[39] Nollen EA , Salomons FA , Brunsting JF , van der Want JJ , Sibon OC and Kampinga HH (2001) Dynamic changes in the localization of thermally unfolded nuclear proteins associated with chaperone‐dependent protection. Proc Natl Acad Sci U S A 98, 12038–12043. 11572931 10.1073/pnas.201112398 PMC59763

[40] Vessey JP , Vaccani A , Xie Y , Dahm R , Karra D , Kiebler MA and Macchi P (2006) Dendritic localization of the translational repressor Pumilio 2 and its contribution to dendritic stress granules. J Neurosci 26, 6496–6508. 16775137 10.1523/JNEUROSCI.0649-06.2006 PMC6674044

[41] Mateju D , Franzmann TM , Patel A , Kopach A , Boczek EE , Maharana S , Lee HO , Carra S , Hyman AA and Alberti S (2017) An aberrant phase transition of stress granules triggered by misfolded protein and prevented by chaperone function. EMBO J 36, 1669–1687. 28377462 10.15252/embj.201695957 PMC5470046

[42] Beaudoin S , Goggin K , Bissonnette C , Grenier C and Roucou X (2008) Aggresomes do not represent a general cellular response to protein misfolding in mammalian cells. BMC Cell Biol 9, 59. 18937858 10.1186/1471-2121-9-59 PMC2576168

[43] Yasuda S , Tsuchiya H , Kaiho A , Guo Q , Ikeuchi K , Endo A , Arai N , Ohtake F , Murata S , Inada T *et al*. (2020) Stress‐ and ubiquitylation‐dependent phase separation of the proteasome. Nature 578, 296–300. 32025036 10.1038/s41586-020-1982-9

[44] Fu A , Cohen‐Kaplan V , Avni N , Livneh I and Ciechanover A (2021) p62‐containing, proteolytically active nuclear condensates, increase the efficiency of the ubiquitin‐proteasome system. Proc Natl Acad Sci U S A 118, e2107321118. 34385323 10.1073/pnas.2107321118 PMC8379982

[45] Alberti S , Gladfelter A and Mittag T (2019) Considerations and challenges in studying liquid‐liquid phase separation and biomolecular condensates. Cell 176, 419–434. 30682370 10.1016/j.cell.2018.12.035 PMC6445271

[46] Romero P , Obradovic Z , Li X , Garner EC , Brown CJ and Dunker AK (2001) Sequence complexity of disordered protein. Proteins 42, 38–48. 11093259 10.1002/1097-0134(20010101)42:1<38::aid-prot50>3.0.co;2-3

[47] Elbaum‐Garfinkle S (2019) Matter over mind: liquid phase separation and neurodegeneration. J Biol Chem 294, 7160–7168. 30914480 10.1074/jbc.REV118.001188 PMC6509495

[48] Shin Y and Brangwynne CP (2017) Liquid phase condensation in cell physiology and disease. Science 357, eaaf4382. 28935776 10.1126/science.aaf4382

[49] Molliex A , Temirov J , Lee J , Coughlin M , Kanagaraj AP , Kim HJ , Mittag T and Taylor JP (2015) Phase separation by low complexity domains promotes stress granule assembly and drives pathological fibrillization. Cell 163, 123–133. 26406374 10.1016/j.cell.2015.09.015 PMC5149108

[50] Banani SF , Lee HO , Hyman AA and Rosen MK (2017) Biomolecular condensates: organizers of cellular biochemistry. Nat Rev Mol Cell Biol 18, 285–298. 28225081 10.1038/nrm.2017.7 PMC7434221

[51] Jain S , Wheeler JR , Walters RW , Agrawal A , Barsic A and Parker R (2016) ATPase‐modulated stress granules contain a diverse proteome and substructure. Cell 164, 487–498. 26777405 10.1016/j.cell.2015.12.038 PMC4733397

[52] Itoh G , Kanno S , Uchida KS , Chiba S , Sugino S , Watanabe K , Mizuno K , Yasui A , Hirota T and Tanaka K (2011) CAMP (C13orf8, ZNF828) is a novel regulator of kinetochore‐microtubule attachment. EMBO J 30, 130–144. 21063390 10.1038/emboj.2010.276 PMC3020106

[53] Jiang H , He X , Wang S , Jia J , Wan Y , Wang Y , Zeng R , Yates J 3rd , Zhu X and Zheng Y (2014) A microtubule‐associated zinc finger protein, BuGZ, regulates mitotic chromosome alignment by ensuring Bub3 stability and kinetochore targeting. Dev Cell 28, 268–281. 24462186 10.1016/j.devcel.2013.12.013 PMC3927447

[54] Sauer G , Korner R , Hanisch A , Ries A , Nigg EA and Sillje HH (2005) Proteome analysis of the human mitotic spindle. Mol Cell Proteomics 4, 35–43. 15561729 10.1074/mcp.M400158-MCP200

[55] Skop AR , Liu H , Yates J 3rd , Meyer BJ and Heald R (2004) Dissection of the mammalian midbody proteome reveals conserved cytokinesis mechanisms. Science 305, 61–66. 15166316 10.1126/science.1097931 PMC3679889

[56] Mohapatra S and Wegmann S (2023) Biomolecular condensation involving the cytoskeleton. Brain Res Bull 194, 105–117. 36690162 10.1016/j.brainresbull.2023.01.009

[57] Feric M , Vaidya N , Harmon TS , Mitrea DM , Zhu L , Richardson TM , Kriwacki RW , Pappu RV and Brangwynne CP (2016) Coexisting liquid phases underlie nucleolar subcompartments. Cell 165, 1686–1697. 27212236 10.1016/j.cell.2016.04.047 PMC5127388

