# Supplementary figures and images for: On subcellular distribution of the zinc finger 469 protein (ZNF469) and observed discrepancy in the localization of endogenous and overexpressed ZNF469
Source: FEBS Open Bio. 2025 Mar 29;15(7):1054–67. doi: 10.1002/2211-5463.70034 (PMC12226416; doi:10.1002/2211-5463.70034)

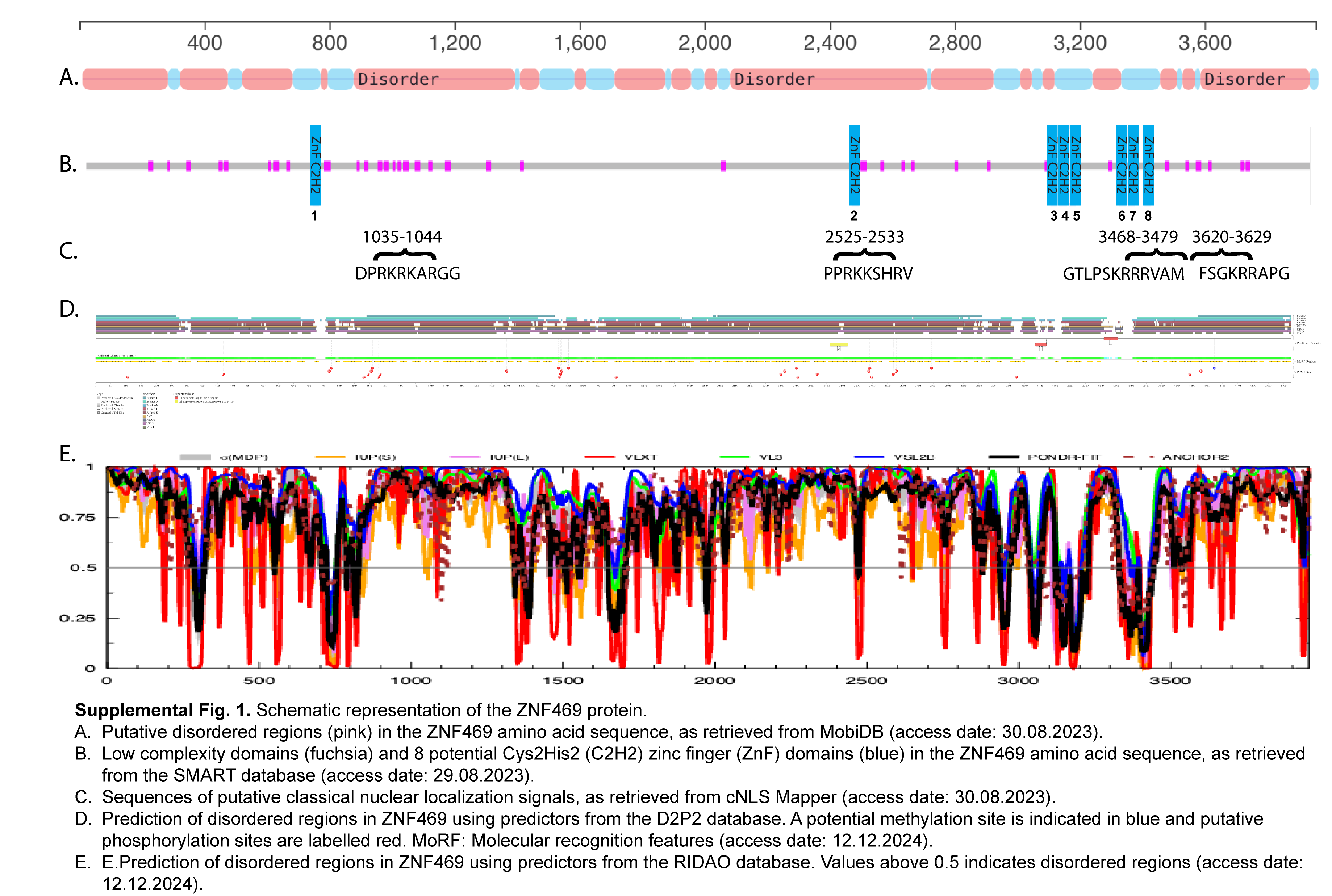

Supplement: Supplementary file 1 — Fig. S1 Schematic representation of the ZNF469 protein with predictions of disordered regions. [file FEB4-15-1054-s001.tiff]

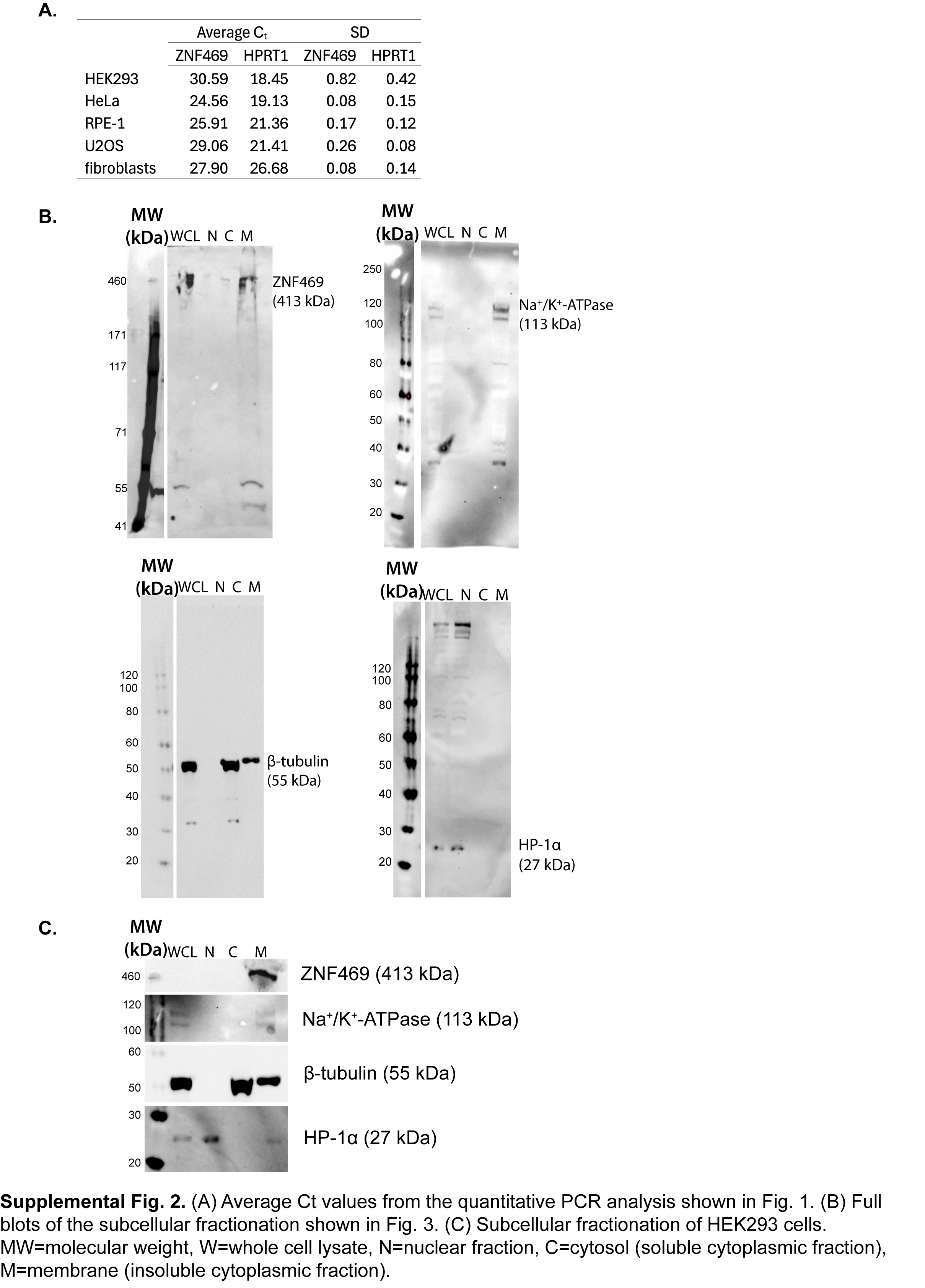

Supplement: Supplementary file 2 — Fig. S2 (A) Average Ct values from the quantitative PCR analysis shown in Fig. 1. (B) Full blots of the subcellular fractionation shown in Fig. 3. (C) Subcellular fractionation of HEK293 cells. [file FEB4-15-1054-s007.tiff]

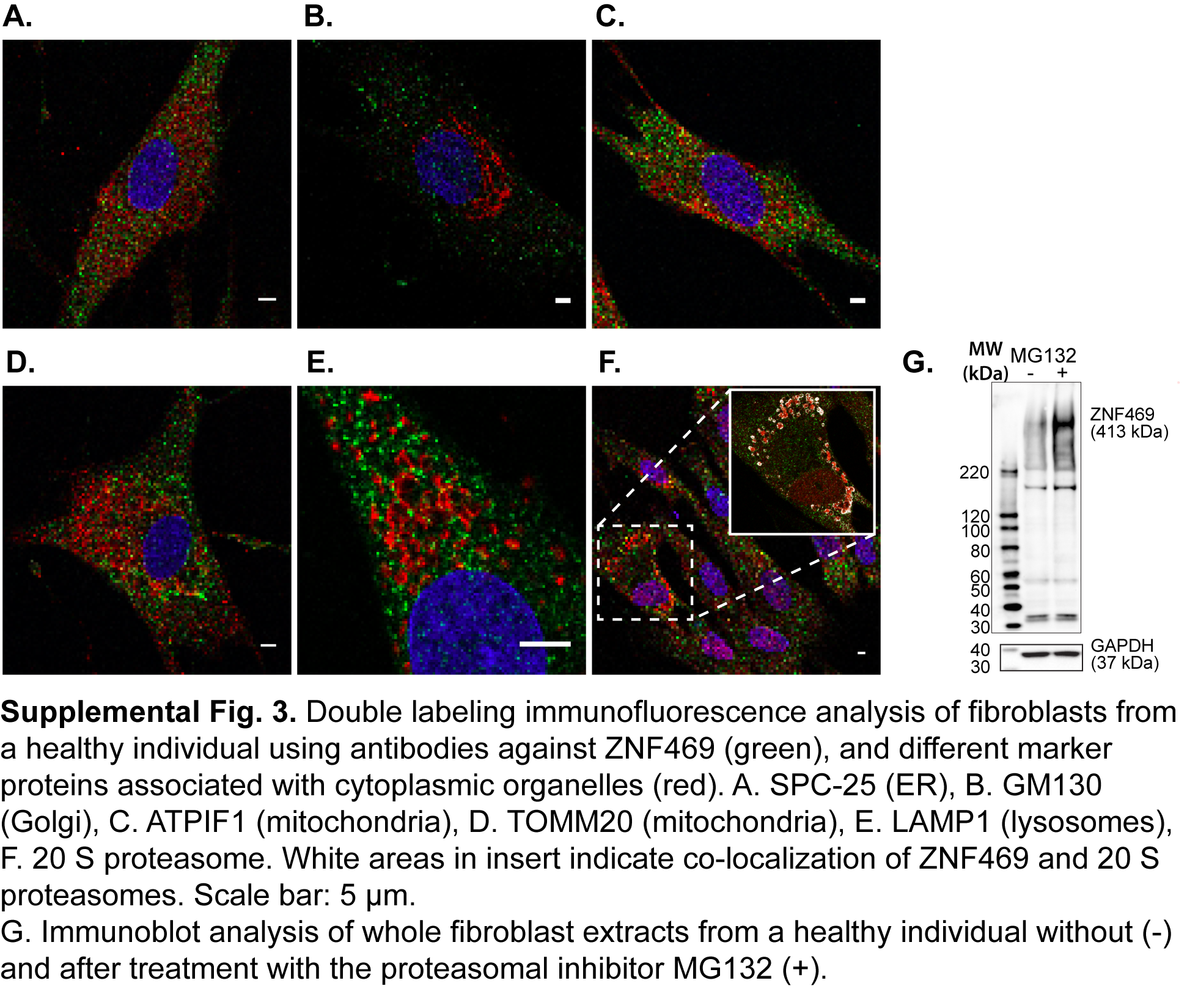

Supplement: Supplementary file 3 — Fig. S3 (A–F) Immunofluorescence analysis of fibroblasts using antibodies against ZNF469 and proteins associated with cytoplasmic organelles. (G) Immunoblot analysis of fibroblast extracts without and after treatment with MG132. [file FEB4-15-1054-s010.tiff]

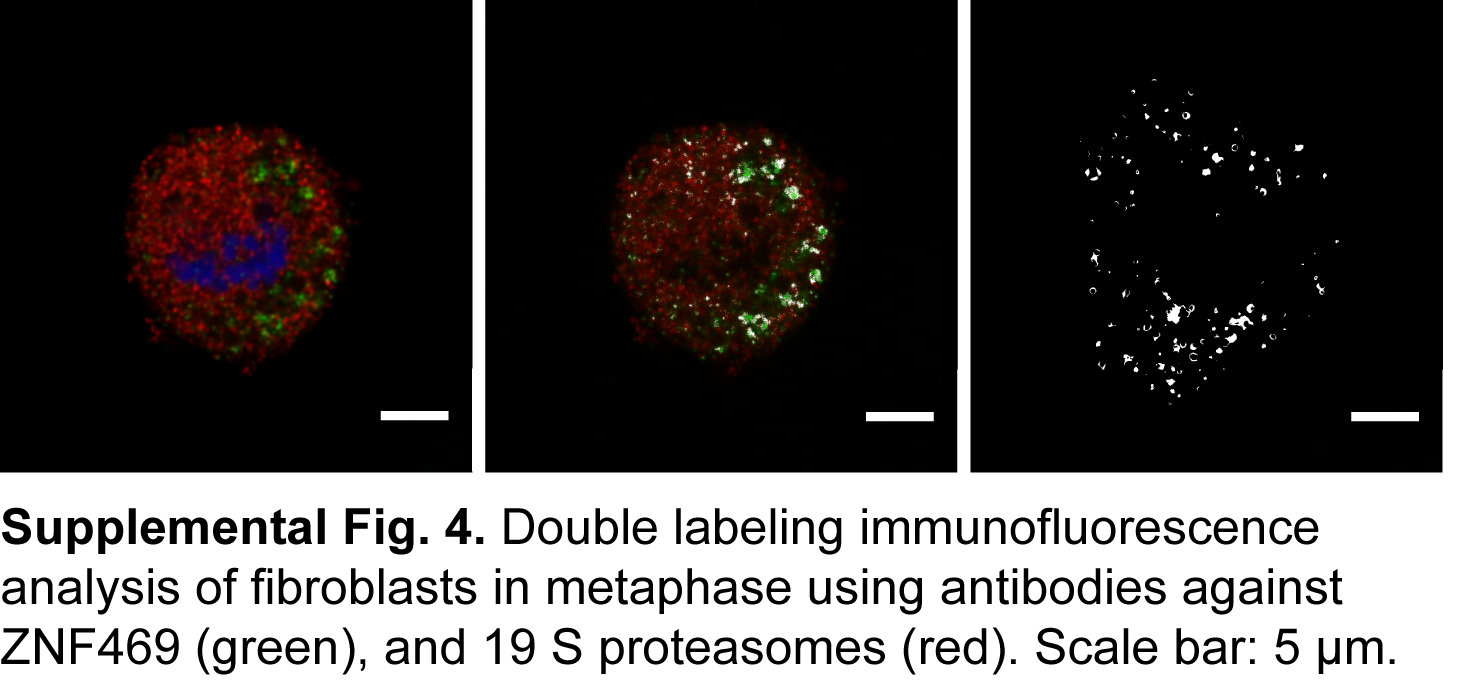

Supplement: Supplementary file 4 — Fig. S4 Immunofluorescence analysis of fibroblasts in metaphase using antibodies against ZNF469 and 19S proteasomes. [file FEB4-15-1054-s003.tif]

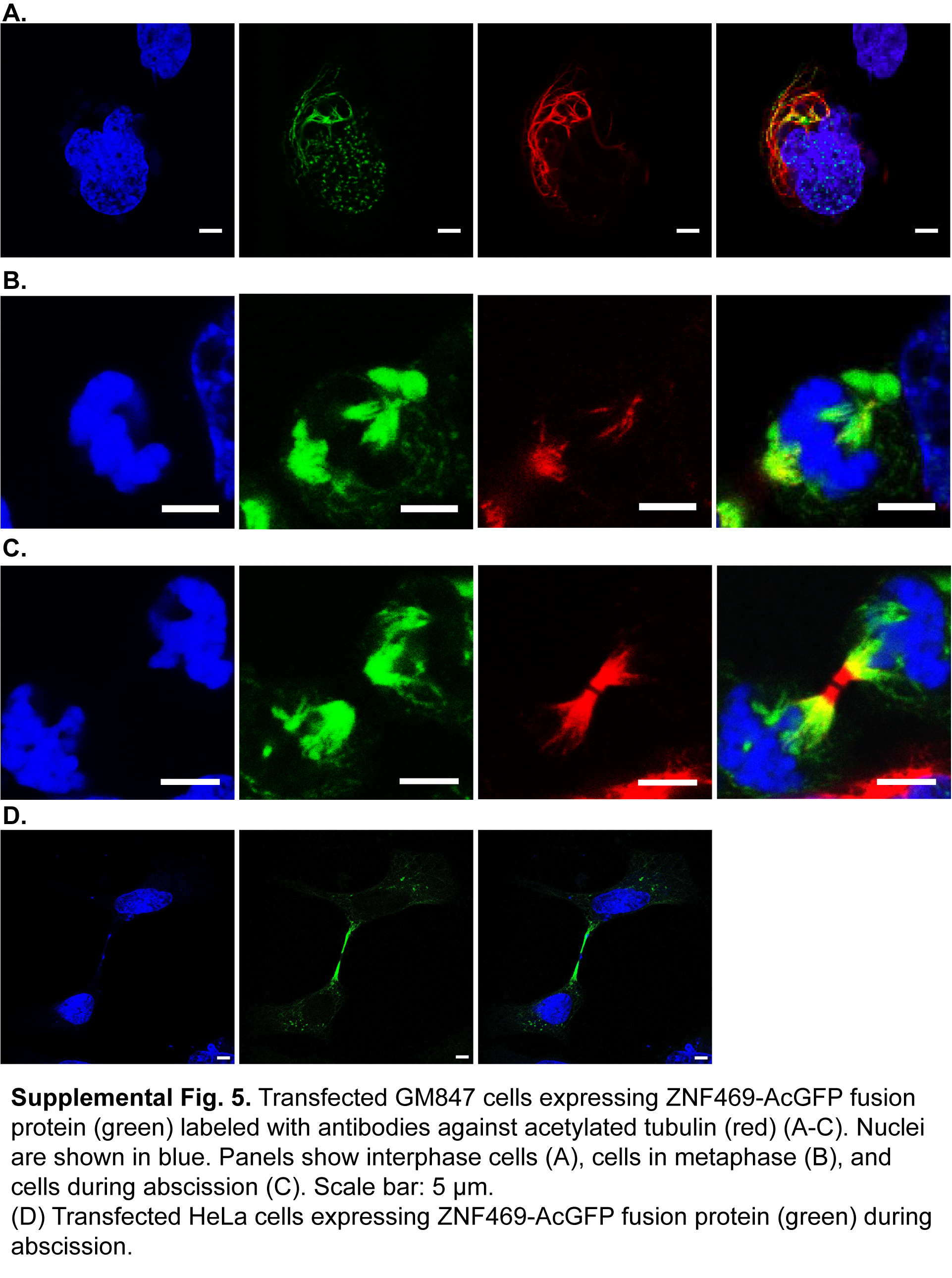

Supplement: Supplementary file 5 — Fig. S5 (A–C) Transfected GM847 cells expressing ZNF469‐AcGFP fusion protein labeled with antibodies against acetylated tubulin. (D) Transfected HeLa cells expressing ZNF469‐AcGFP fusion protein during abscission. [file FEB4-15-1054-s002.tiff]

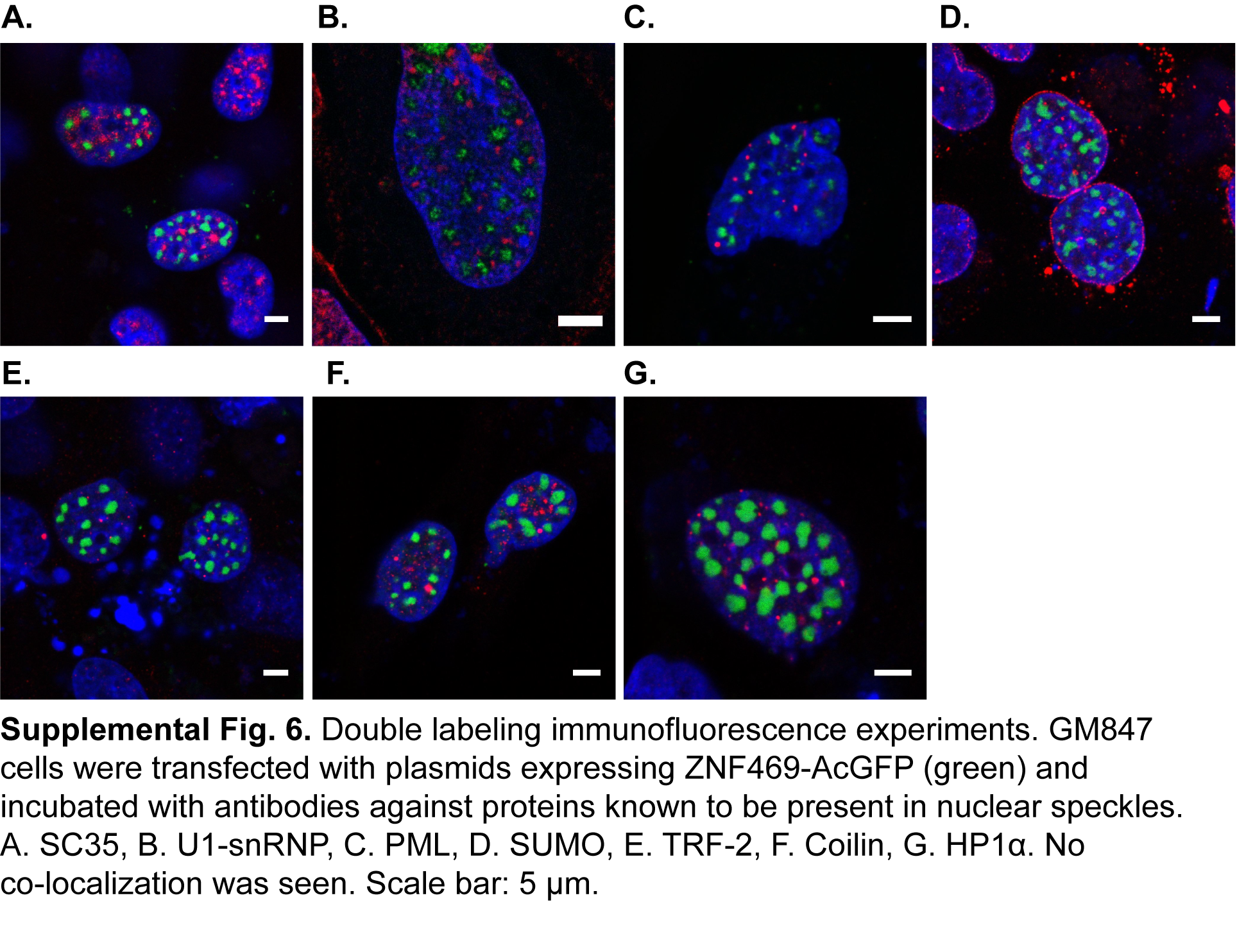

Supplement: Supplementary file 6 — Fig. S6 Immunofluorescence analysis of GM847 cells transfected with ZNF469‐AcGFP labeled with antibodies against nuclear proteins. [file FEB4-15-1054-s008.tif]

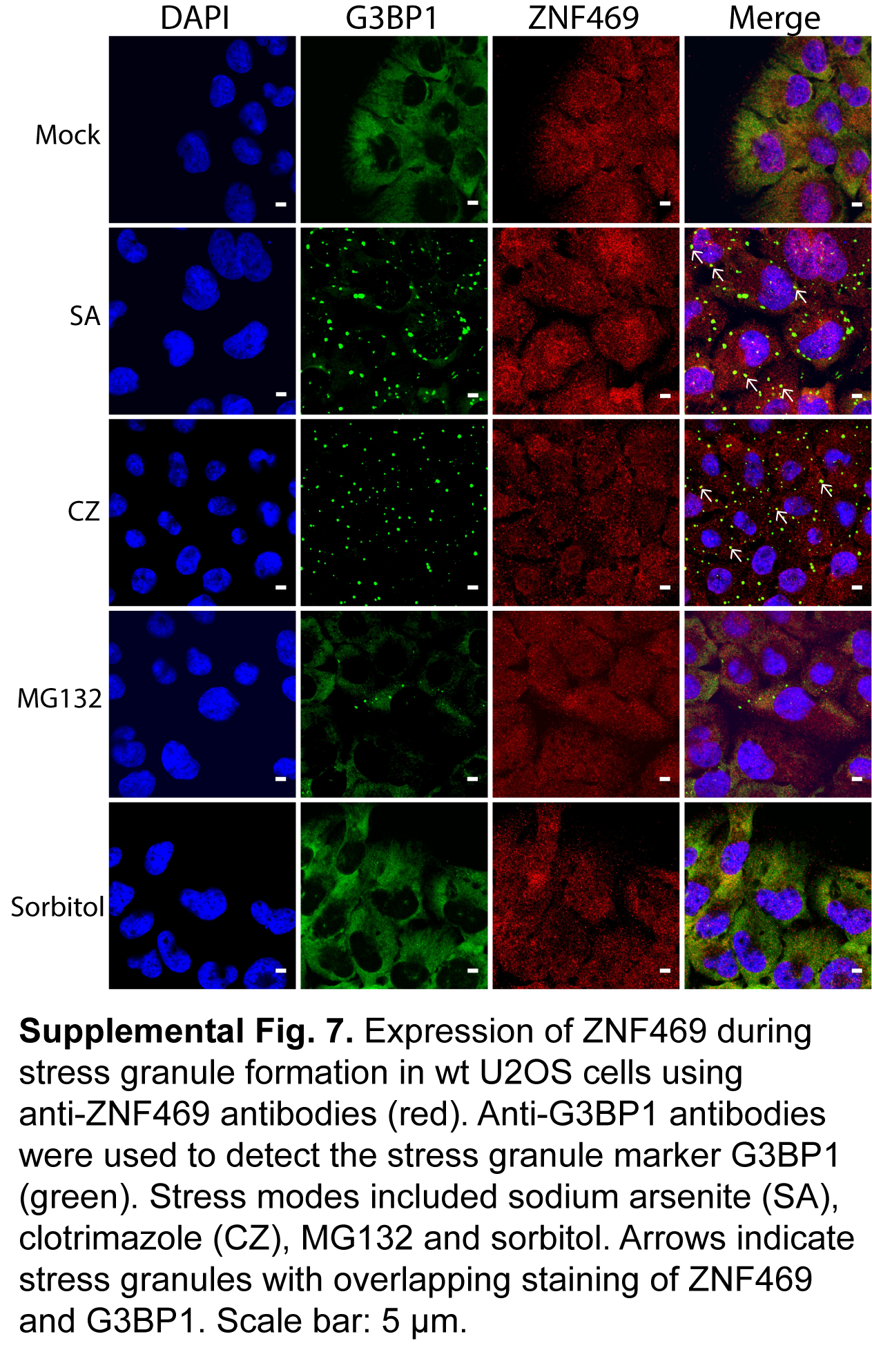

Supplement: Supplementary file 7 — Fig. S7 Expression of ZNF469 during stress granule formation in wt U2OS cells using anti‐ZNF469 antibodies. [file FEB4-15-1054-s009.tiff]

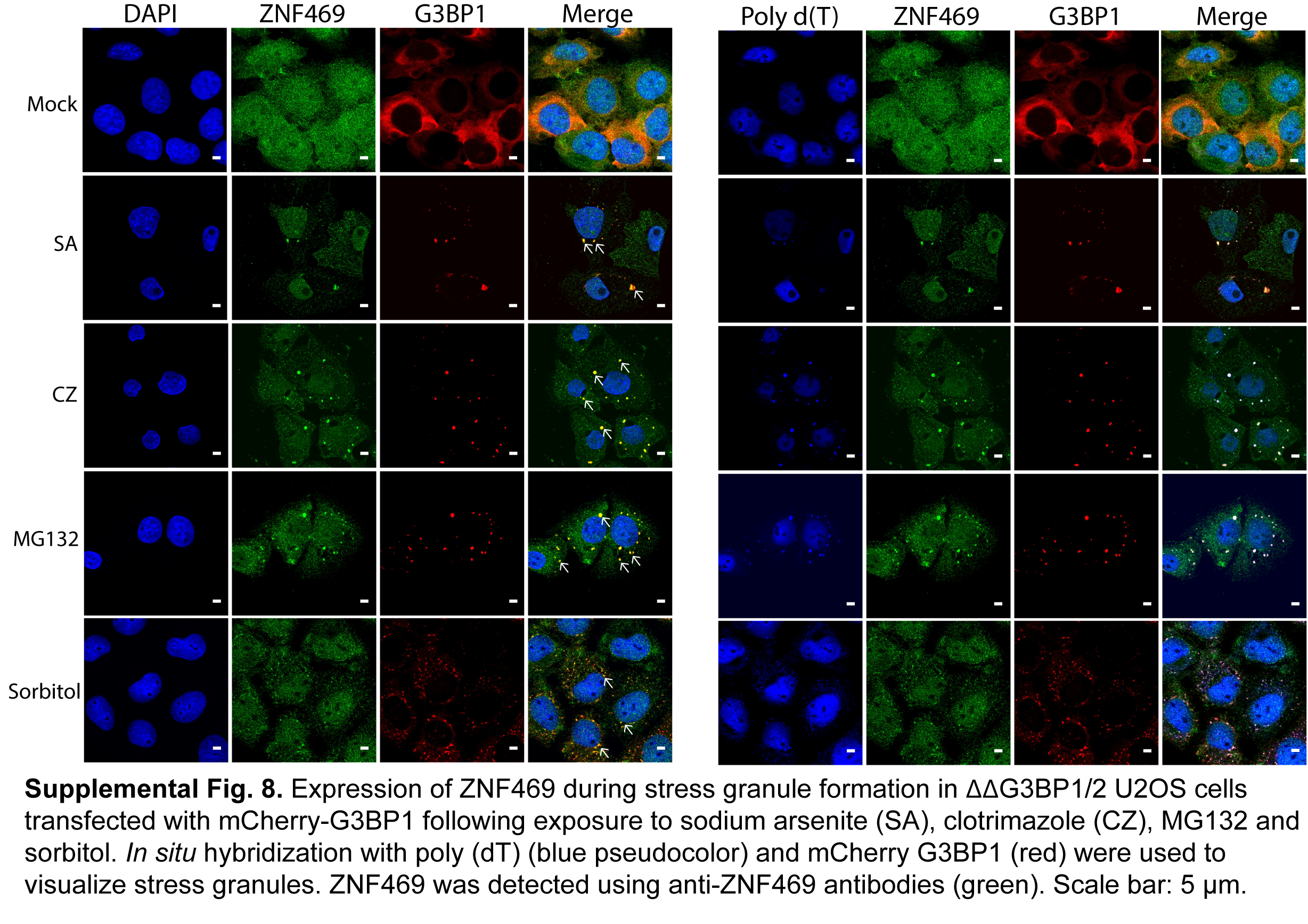

Supplement: Supplementary file 8 — Fig. S8 Expression of ZNF469 during stress granule formation in ΔΔG3BP1/2 U2OS cells transfected with mCherry‐G3BP1. [file FEB4-15-1054-s005.tiff]
